# Controlling the Electromagnetic Field Confinement with Metamaterials

**DOI:** 10.1038/srep37739

**Published:** 2016-11-25

**Authors:** Jordi Bonache, Gerard Zamora, Ferran Paredes, Simone Zuffanelli, Pau Aguilà, Ferran Martín

**Affiliations:** 1 Departament d’Enginyeria Electronica, Universitat Autonoma de Barcelona, 08193 Bellaterra, Spain

## Abstract

The definition of a precise illumination region is essential in many applications where the electromagnetic field should be confined in some specific volume. By using conventional structures, it is difficult to achieve an adequate confinement distance (or volume) with negligible levels of radiation leakage beyond it. Although metamaterial structures and metasurfaces are well-known to provide high controllability of their electromagnetic properties, this feature has not yet been applied to solve this problem. We present a method of electromagnetic field confinement based on the generation of evanescent waves by means of metamaterial structures. With this method, the confinement volume can be controlled, namely, it is possible to define a large area with an intense field without radiation leakage. A prototype working in the microwave region has been implemented, and very good agreement between the measurements and the theoretical prediction of field distribution has been obtained.

The control of electromagnetic field distribution in space is of fundamental importance in all applications where an interaction between the field and any actuator, transponder, sensor or sample is required. However, the definition of a large bounded illumination region with strong field levels is troublesome by using conventional structures due to the presence of energy leakage in form of radiation, especially when the dimension of said region is comparable to the wavelength of the electromagnetic waves generated by the source^1^,^2^. To solve these difficulties, evanescent wave coupling can be considered, since the field intensity of evanescent modes decreases exponentially with distance and hence these modes do not radiate. Some examples of applications can be found, among others, in the total internal reflection fluorescence microscope^3^,^4^,^5^,^6^, optical fiber coupling^7^, prism coupling^7^,^8^,^9^, excitation of surface plasmon polaritons^10^,^11^, wireless energy transfer^12^, etc. Some of these applications demand highly confined fields (for example in microscopy), while in other applications a significant confinement distance is desired (e.g., in wireless energy transfer). Nevertheless, the confinement of these waves depends directly on the materials used for the implementation of the coupler and the surrounding media, resulting in a poor controllability of the field decay with distance. Conversely, it is well established that metamaterial wave-guiding structures^13^,^14^,^15^,^16^,^17^,^18^ (i.e., periodic structures with the period much smaller than the wavelength) and metasurfaces offer high controllability of the field distribution regardless of the materials composing the structure^19^,^20^,^21^,^22^. However, to the author’s knowledge such controllability has not been exploited in order to obtain a specific field confinement region optimized for a certain application.

In other technologies, such as Radio Frequency Identification (RFID) in the ultra-high frequency (UHF) band, the lack of control in field confinement keeps many potential applications unexplored^23^. Indeed, the main aim of this technology is the identification of objects up to several meters away from the reader by means of a radio signal. These objects must incorporate an RFID tag which is composed of an antenna (usually a dipole antenna, i.e., linearly polarized) and an integrated circuit. The antenna enables the communication with the reader, while the integrated circuit stores information and identifies the object. The current regulations for UHF-RFID impose the maximum level of the power density sent by the reader, whereas there is no restriction on the field polarization, which could be defined depending on the application^23^. These two parameters, in addition to the distance between tag and reader and the characteristics of the tag antenna (i.e., antenna gain and matching with the chip), determine the total power received by the integrated circuit of the tag, and hence, the maximum reading distance.

However, reading the UHF-RFID tags at small (and controllable) distances may be interesting in applications such as Points of Sale at shops and stores. For that purpose, specific readers operating in the near field, rather than in the radiative mode, must be designed. With such readers, the objects inside the small (and predefined) reading distance are detected, whereas those outside this region are ignored, avoiding thus undesired readings. Note that with these near-field readers, the functionality of UHF-RFID tags is twofold: object identification and tracking at large distances (with conventional radiative-mode readers), and detection at low distances (by using the proposed field confinement readers), as is required in Points of Sale and in other applications, such as access control, inventory at short distances, etc. However, since the frequency of operation of UHF-RFID readers is near the GHz range (915 MHz in USA and 867 MHz in Europe) the achievable field confinement distances in typical-non-radiative devices are limited to a few centimeters^24^ (a small fraction of the wavelength). Some solutions are proposed to operate under this reduced reading distance by means of an inductive or capacitive coupling between the tag and the reader^25^,^26^,^27^,^28^. Nevertheless, for most applications, such as the near field identification of objects in the Point of Sale at shops and stores, these distances could be inadequate. The use of magnetic coils with dimensions comparable to the wavelength was proposed to increase this reading distance^2^,^29^. Although this configuration produces significant radiation levels, the use of special tags sensitive to the magnetic field may limit the reading distance to several centimeters due to the strong field required by these tags, which is great enough only in the vicinity of the interrogating coil. Nevertheless, the strong radiation leakage produced by the coil makes this technology incompatible with the use of standard tags based on dipole antennas, which would be detected up to several meters away from the reader.

In this paper, we present a method for generating an evanescent wave with controllable levels of confinement, which is applicable from radio to optical wavelengths. Unlike previous works, where the control of the field characteristics is achieved by means of bidimensional structures, we introduce for the first time a planar one-dimensional Metamaterial Transmission Line to illuminate some specific volume of the space with an evanescent field. Thanks to the possibility to simultaneously and accurately tailor the phase velocity and Bloch impedance at wish with these structures, the generation of evanescent waves with the desired level of confinement is possible.

## Results

The proposed method consists in designing a one-dimensional metamaterial guiding structure that propagates a surface wave mode with controllable levels of field decay in the surrounding medium. For this purpose, the wave number *β* of the guiding mode must be tailored to obtain the required exponential decay factor *α*_*y*_ according to (see Supplementary Information):


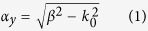


where *k*_0_ = *ω*/*c*_0_, *c*_0_ being the phase velocity of the wave in the surrounding medium.

**Figure 1:**
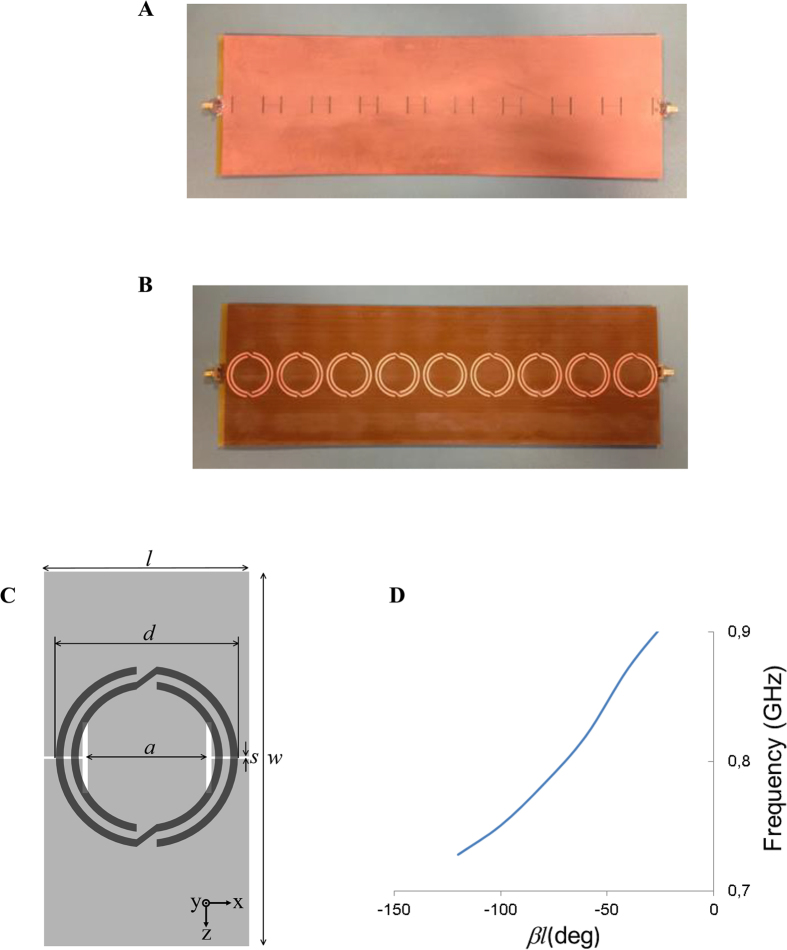
Metamaterial structure functional at the UHF frequency band. Photograph of **(A)** top layer and **(B)** bottom layer of the fabricated device, which presents a total length of 31 cm (0.9 times the free-space wavelength at 867 MHz). **(C)** Unit cell of the proposed structure. The light and dark grey colors represent copper traces in the top and bottom layer, respectively. The dimensions of the unit cell are *l* = 34.57 mm (0.1 times the free-space wavelength at 867 MHz) and *w* = 100 mm (0.29 times the free-space wavelength at 867 MHz), the external ring diameter is *d* = 32 mm, the width of the rings and the separation between them are 1.35 mm, the width of the longitudinal slot is *s* = 0.2 mm, the length and width of the transversal slot are 12.55 mm and 0.94 mm, respectively, and the width of the metallic connections in the slot is *a* = 21 mm. **(D)** Dispersion relation of the structure retrieved from the measured S parameters. Negative sign in *βl* denotes a left-handed mode operation.

To verify the functionality of the proposed method, we implemented a device designed to operate in the UHF band, specifically in the European RFID band (867 MHz) (Fig. 1). The design is based on the one-dimensional metamaterial periodic structure proposed in ref. 30. As shown in that work, a symmetric CPW with a wide central strip exhibits similar propagation characteristics than the slot line resulting by removing one half of the CPW structure. This allows designing the proposed device taking advantage of the equivalent circuit model of the CPW structure. For the design proposed in the present work it was found a dimension of *w* = 100 mm (0.29 times the free-space wavelength at 867 MHz) along the *z*-axis to be sufficiently wide. The device, formed by 9 unit cells, was implemented by using two levels of metallization (copper, 35 μm thickness) on a planar low-loss dielectric material (Nelco N4350 with *ε*_*r*_ = 3.5, tan*δ* = 0.0065 and *h* = 0.5 mm). Figure 1C shows the unit cell of the structure. The top layer (copper traces depicted in light grey) contains a slot transmission line with a metallic connection within the slot, whereas the bottom layer (copper traces depicted in dark grey) contains a Non Bianisotropic Split Ring Resonator^31^,^32^ (NBSRR) magnetically coupled to the slot line. In this structure, the metallic connections (of width *a*) within the slots are used to adjust the value of the effective dielectric permittivity (*ε*), whereas the value of the effective magnetic permeability (*μ*) is tailored by means of the NBSRRs. The control of both parameters (*ε* and *μ*) allows us to force the required value of the wavenumber *β* (see Supplementary Information) to obtain the value of *α*_*y*_ dictated by the considered application. In our case, the attenuation distance (1/*α*_*y*_) was set to 8.7 cm (or, equivalently, 0.25 times the free-space wavelength at 867 MHz, corresponding to an attenuation of 1 dB/cm), and to achieve this value, the electrical length of the unit cell was forced to be *βl* = −42.5° (−1.15 times the electrical length in free-space *k*_0_*l*) (Fig. 1D). The negative sign of *β* means that the device operates in the left-handed (or backward wave) frequency band (where *μ* and ε are simultaneously negative). According to Equation (1), a solution in the right-handed band does also exist, corresponding to a positive value of the wave number *β* (with *μ* and ε simultaneously positive). In this structure, the left-handed band appears at lower frequencies^30^ and, therefore, operation at the desired frequency (867 MHz) within the right-handed band would require an increase of the device size, with respect to working at the left-handed band. On account of this, operation in the left-handed band was chosen for homogenization purposes. By properly adjusting the ratio between the effective permeability and permittivity, the Bloch impedance of the structure (equivalent to the characteristic impedance of an ordinary transmission line, and given by 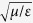) was forced to be 50 Ω at 867 MHz.

The field distribution around the structure is determined by the magnetic current of the slot line (which generates a symmetrical field distribution in the upper and lower side of the device) in addition to the contribution of the NBSRRs. Since the dimensions of the NBSRRs are small in terms of wavelength, the contribution to the field of these resonators will be concentrated around their geometry. This contribution to the field surrounding the metamaterial can be accounted for the higher order Floquet modes. However, since these modes generate more confined waves at the operating frequency (their wavelength is shorter than the fundamental Floquet mode), at some distance from the surface their contribution will be negligible. This device presents a current distribution that maximizes the electric field in the *z*-direction in most of the space around the structure (TE^*x*^-polarization, as expected from the field distribution of a slot line propagating in the *x*-direction). This ensures reading of tags oriented along the *z*-direction. Although it is a unidimensional structure, the field distribution in the vicinity of the symmetry plane of the device (*xy*-plane containing the slot of the host line) is uniform with respect to the *z*-coordinate. A more detailed description of the electric field distribution around the metamaterial is shown in Section II of the Supplementary Information.

**Figure 2:**
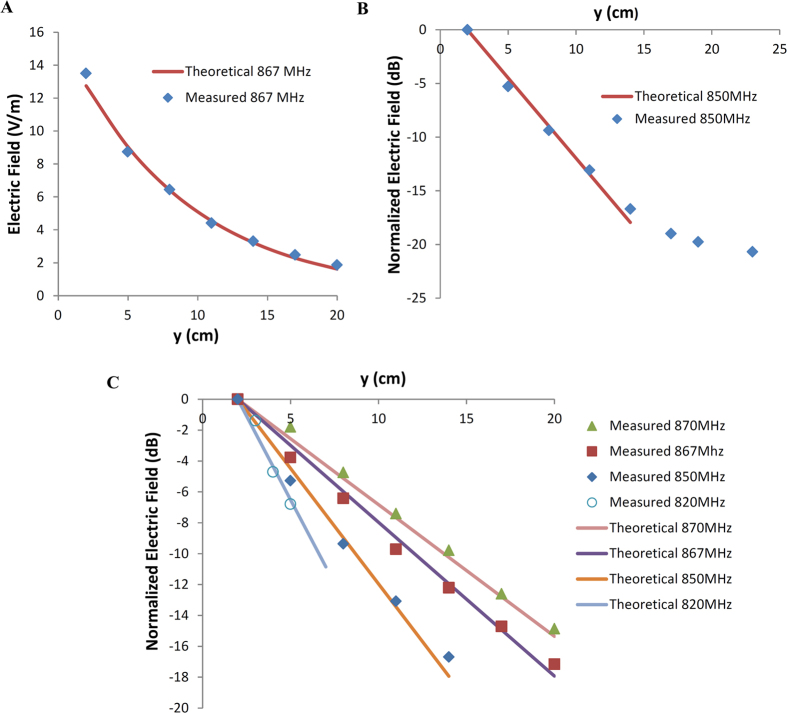
Experimental results. Measured (discrete points) and theoretical prediction (continuous line) of the electric field in the *z*-direction versus distance. **(A)** Results at 867 MHz expressed in linear. The theoretical calculation was obtained using the exponential decay factor of the field given by Equation (1) and taking as amplitude the first measured value (2 cm away from the structure) **(B)** Results at 850 MHz expressed in dB. **(C)** Comparison of the field decay at different frequencies in dB.

Figure 2A shows the measured electric field in the *z*-direction (obtained by means of the experimental setup and the measurement procedure described in the Supplementary Information) versus the distance from the metamaterial transmission line measured along the *y*-direction. In the same picture, the theoretical prediction of the exponential decay factor of the electric field given by Equation (1) is represented. Measurement data and theory exhibit very good agreement. We measured the field at several frequencies in order to verify the behavior of the field decay according to Equation (1). Although the Bloch impedance of the structure was imposed to the desired value at 867 MHz, the field decay fits reasonably well with theory at other frequencies. Figure 2B shows the measured and theoretical field levels expressed in dB (normalized to the field level at 2 cm away from the surface of the metamaterial structure) at 850 MHz. From the dispersion relation of Fig. 1D, the electrical length of the unit cell is *βl* = −49° at this frequency, corresponding to 1/*α*_*y*_ = 5.8 cm and implying an attenuation of 1.5 dB/cm. In this case, the exponential decay results in a straight line in the logarithmic plot. Due to the mismatch between the metamaterial structure and the reference impedance (reflection coefficient is around −10 dB at 850 MHz), some levels of radiation are expected^1^. This may cause some deviation of the measured electric field from the theoretical curve above some distance (beyond 14 cm in Fig. 2B). Nevertheless, in the region where the radiated field is negligible the field decay is perfectly predicted by theory. Figure 2C depicts the normalized electric field versus distance for 820 MHz, 850 MHz, 867 MHz and 870 MHz. Only the region dominated by the confined wave is represented (lower frequencies result in smaller linear part in the logarithmic plot since the matching with the reference impedance is worse). The agreement between the measured and theoretical prediction is very good as well.

Simulations and measurements of the scattering parameters are provided in the Supplementary Information. The measured insertion losses (4.8 dB at 867 MHz) correspond to an attenuation of 0.15 dB/cm. This result evinces the efficiency of the proposed device in terms of power dissipation.

## Discussion

From the application point of view, this structure can be directly applied to an UHF-RFID nearfield reader since a zone where the tag can be detected is clearly defined. Commercial UHF-RFID tags usually need an electric field around 2 V/m for their activation. This field strength is obtained (using an input power of 2 W, see Supplementary Information) at 20 cm from the surface of the proposed device (at 867 MHz). The attenuation of 1 dB/cm ensures a reduction of the power density in a factor of two every 3 cm; hence the tag will be rapidly undetectable beyond this point. On the contrary, the power density increases in a factor of two every 3 cm as the tag approaches the metamaterial transmission line surface, thus ensuring a good detection rate for closer distances. A redesign of the dispersion relation and a proper adjustment of the input power will provide other detection ranges. The application of this field confinement method is not only restricted to the UHF frequency band or to the designed metamaterial structure. It can be used in other frequency ranges where other metamaterial structures or metasurfaces can be implemented, e.g., millimeter wave band or optical frequencies.

## Additional Information

**How to cite this article**: Bonache, J. *et al.* Controlling the electromagnetic field confinement with metamaterials. *Sci. Rep.* **6**, 37739; doi: 10.1038/srep37739 (2016).

**Publisher's note:** Springer Nature remains neutral with regard to jurisdictional claims in published maps and institutional affiliations.

## Supplementary Material

Supplementary Information
