# Supplementary material for: Controlling the Electromagnetic Field Confinement with Metamaterials
Source: Sci Rep. 2016 Nov 25;6:37739. doi: 10.1038/srep37739 (PMC5123569; doi:10.1038/srep37739)
Supplement: Supplementary Information [file srep37739-s1.pdf]

Supplementary Information for "Controlling the Electromagnetic Field Confinement with Metamaterials"

Jordi Bonache, Gerard Zamora, Ferran Paredes, Simone Zuffanelli, Pau Aguila and Ferran Martin

Departament d'Enginyeria Electronica, Universitat Autonoma de Barcelona, 08193 Bellaterra, Spain

I. METHODS

Fabrication of the device

The proposed device was fabricated by means of an *LPKF ProtoMat H100* drilling machine.

Experimental setup

The fabricated device was excited by means of a pure tone created by a signal generator and transferred through a coaxial cable ended with a 3.5 mm SMA connector (placed between the two metallic strips of the top layer). By using a power amplifier, a total power of 2 W was delivered to the structure. The device was terminated with a 50 Ω resistive load. Electric field measurements were taken by means of an electric field probe (*Wavecontrol EF Cube*, calibrated by *Wavecontrol S.L.*). Fig. S1 shows a schematic of the experimental setup used for the electric field measurements.

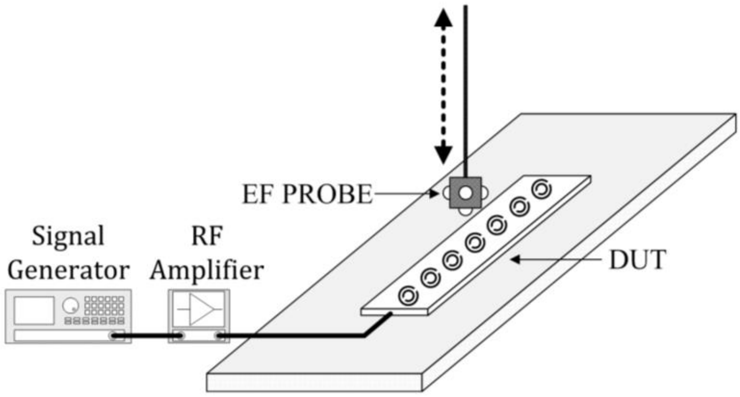

Fig. S1. Schematic of the experimental setup used for the electric field measurements.

Measurements

Electric field measurements were carried out locating the electric field probe at a distance of $x = 10$ cm (0.29 times the free-space wavelength at 867 MHz) from the feeding point of the structure, varying the distance from the metamaterial transmission line along the y-direction (within the xy-plane containing the slot of the host line). The field probe used in the measurement process presents a lateral dimension of roughly 5 cm, precluding obtaining electric field data at very short distances from the device surface. For this reason the measured data accounts for the fundamental Floquet mode only.

Measurements of the scattering parameters were obtained by means of the *Agilent PNA N5221A* network analyzer.

Numerical calculations

Full wave simulations were carried out by means of the commercial software *CST Microwave Studio* for the electric field around the device, and the *Keysight ADS Momentum* commercial software for the scattering parameters of the proposed structure.

II. PRINCIPLE OF OPERATION OF THE DEVICE

It is well known that at the interface between two different media the tangential component of the wave vector $\vec{k}$ along such interface must be continuous. This vector is in general complex [1, 2] and its components are related by the separability condition of the wave equation. For the case of a medium surrounding the metamaterial structure, such relation in rectangular coordinates gives

$$k_x^2 + k_y^2 + k_z^2 = k_0^2 \tag{S1}$$

where $k_i = \beta_i - j\alpha_i$, with β_i the phase constant and α_i the attenuation of the wave along the i-direction, and $k_0^2 = (\omega/c_0)^2$, c_0 being the phase velocity of the wave in the surrounding medium. For simplicity we assume a mode that has no z variation ($k_z = 0$) and propagates within the metamaterial structure along the x-direction. To ensure continuity of the tangential components of the wave vector, k_x must match the wave vector inside the metamaterial (assuming the metamaterial is periodic with the period much smaller than the wavelength), i.e., $k_x = \beta - j\alpha$, where β and α are the phase and attenuation constants of the guided mode, respectively. It follows from equation (S1) that

$$k_y = \pm\sqrt{k_0^2 - (\beta - j\alpha)^2} \tag{S2}$$

There are two different modes of operation for the wave that propagates within the metamaterial structure, the radiative and the surface wave mode. The former, which gives rise to complex values of k_y, is based on a wave that propagates along the guiding structure gradually leaking out a small amount of energy in the form of coherent radiation, which implies an attenuation of the guided signal accounted by the parameter α. The latter consists of a wave that propagates in the direction of the guiding device (x-direction) and decays vertically (y-direction), which means that the phase constant $\beta_y = 0$ and $k_y = -j\alpha_y$. Since there is no radiation, the signal is propagated without attenuation inside the metamaterial structure (provided that losses in the guiding structure are negligible) and α vanishes.

Recalling the general solution of plane wave propagation in free space, the total field in the surrounding medium can be expressed according to:

$$A_i(x,y,t) = A_i e^{-j\vec{k}\cdot\vec{r}} e^{j\omega t} = A_i e^{-jk_x x} e^{-jk_y y} e^{j\omega t} \tag{S3}$$

where A_i corresponds to any component of the total electromagnetic field propagating in the surrounding medium. For the case of the radiative operation, the wave vector $\vec{k}$ is complex, giving rise to inhomogeneous waves when placed in equation (S3) (leaky waves). Commonly, the attenuation constant in this mode of operation is small enough to be neglected in the calculation of the angle of maximum radiation intensity. Under this assumption, the consideration of just the real part of the wave vector is enough for the estimation of the angle of emission (see Fig. S2a) [3-8].

For the case of the surface wave mode, equation (S2) becomes:

$$k_y = \pm\sqrt{k_0^2 - \beta^2} \tag{S4}$$

So, by properly adjusting the value of β^2 (depending on the sign of the radicand in equation (S4)), purely-imaginary values of k_y are obtained. The wave number β can be adjusted by appropriately choosing the effective dielectric permittivity (ε) and the effective magnetic permeability (μ) of the structure [8-12] using $\beta^2 = \omega^2\mu\varepsilon$. Therefore, by designing the metamaterial structure in order to get purely-imaginary values of k_y (i.e., $\beta^2>k^2$) one obtains:

$$A_i(x,y,t) = A_i e^{-j\vec{k}\cdot\vec{r}} e^{j\omega t} = A_i e^{-j\beta x} e^{-\alpha_y y} e^{j\omega t} \quad \text{(S5)}$$

where $\alpha_y = \sqrt{\beta^2 - k_0^2}$ is a positive (ensuring that fields vanish at infinity) real number. That supposes an exponential decrease of the electric and magnetic fields with distance in the broadside direction (y-direction) (see Fig. S2b). Since the value of β can be adjusted in the design stage of the metamaterial structure, it is possible to impose the level of confinement of the electromagnetic field around the structure. Moreover, since radiation is prevented, the dimensions of the structure might be arbitrarily large (increasing the number of unit cells) in order to increase the coverage area, provided that dielectric and conductor losses present in the structure are low enough. The parameter $1/\alpha_y$ can be defined as a characteristic distance d, since it determines the maximum length where the field will be present. Specifically, $5d$ corresponds to an attenuation of 99% with respect to the field at the surface of the device, so it can be considered that the field is confined within this distance. The lower limit for α_y corresponds to a wave number $\beta = k_0$, whereas the upper limit is determined by the maximum achievable value for β. This value is related to the maximum size of the unit cell (i.e., the constituent elements of a periodic metamaterial guiding structure) to ensure that refractive phenomena dominate over dispersion phenomena. It is also called effective homogeneity limit and is given by $p = \lambda_g/4$, λ_g being the guided wavelength [13]. This limit gives rise to the upper bound for the electrical length of a single unit cell at the operating frequency, that is $\beta p = \pi/2$. It follows that the maximum achievable value for β is $\beta_{\max} = \pi/(2l_{\min})$, $l_{\min}$ being the minimum physical length of the unit cell providing an electrical length of $\pi/2$. Typical values for $l_{\min}$, using high dielectric permittivity substrates (e.g., the Rogers RO3010 with dielectric constant $\varepsilon_r = 10.2$ and thickness $h = 1.27$ mm), are in the order of $l_{\min} = \lambda_0/20$ [14]. Therefore, we obtain that $\alpha_y^{\mathrm{MIN}} = 0$ and $\alpha_y^{\mathrm{MAX}} \approx 31/\lambda_0$. Specifically, for the case of the European RFID band (867 MHz) we obtain $\alpha_y^{\mathrm{MAX}} \approx 90\ \mathrm{m}^{-1}$, corresponding to an attenuation of roughly 8 dB/cm. Nevertheless, the level of confinement will be also limited by the spectrum of the propagating mode in k-space. In practice, this representation in k-space will present some spectral width that will produce some radiation if part of this spectrum lays into the fast wave region. This point will be especially important for less confined waves, since the phase velocity of the mode approaches c. The main sources of spectrum broadening will be the physical limitation of the dimension of the device and the presence of losses in the structure, since these phenomena may induce significant discrepancies from the ideal case of a mode described with a single spatial harmonic (which is infinitely long and with constant amplitude).

Figure S2 illustrates the qualitative behavior of a metamaterial guiding structure working in the radiative mode (Fig. S2a) and the surface wave mode (Fig. S2b). In this case the coordinate indexes are chosen to indicate the propagation of the wave in the metamaterial structure according to the x axis and the induced wave in the xy-plane.

a

b

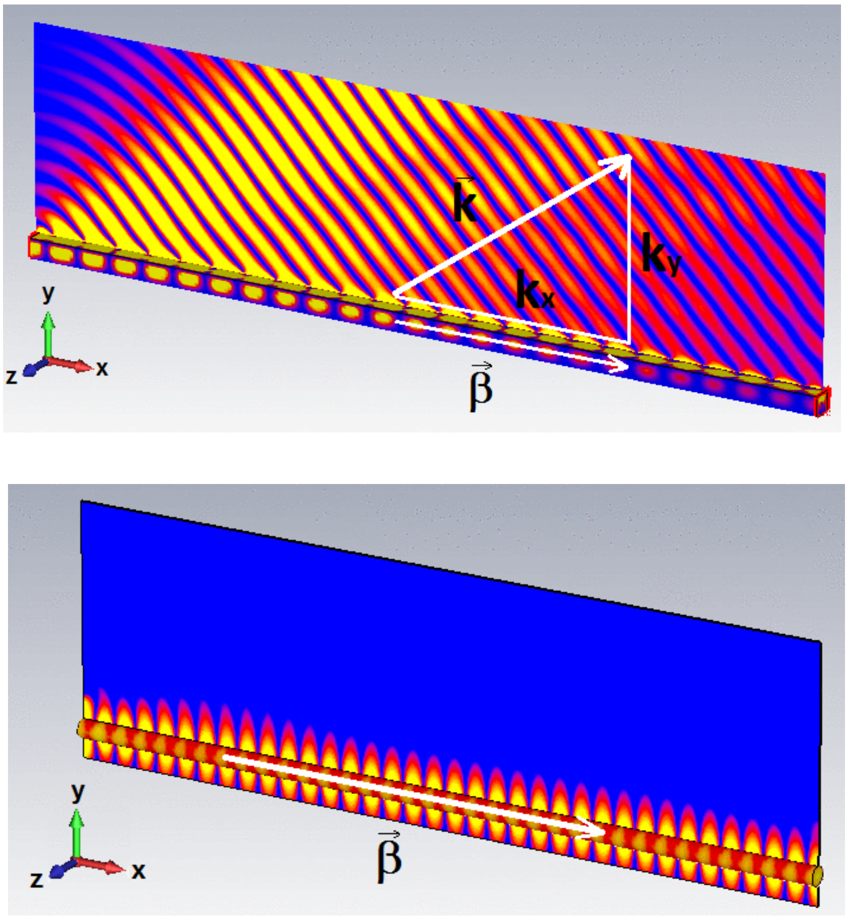

Fig. S2. Field distribution around the metamaterial structure. (a) Radiative mode operation and **(b)** evanescent mode operation, where $\vec{\beta}$ corresponds to the real part of the complex wave vector of the wave traveling through the metamaterial structure, $\vec{k}$ corresponds to the real part of the complex wave vector of the wave propagating in the surrounding medium (induced by the field inside the metamaterial structure) and k_x and k_y correspond to the projection of the longitudinal and transverse components of $\vec{k}$. The picture of the radiative mode was obtained simulating a 16 wavelength long coaxial line with square cross section of 4 cm width filled with a homogeneous material of relative permittivity and permeability $\varepsilon_r = \mu_r = 0.5$ with a slot placed in the top of its geometry to allow radiation. The displayed electric field range level covers up to 100 V/m for an excitation of 1W in a quasi TEM mode at the input port. The picture of the evanescent mode was obtained simulating a 16 wavelength long circular waveguide with 3 cm of radius made with a homogeneous material of $\varepsilon_r = \mu_r = 1.5$. The electric field range level covers up to 100 V/m for an excitation of 1 W in a hybrid mode at the input port. In both cases input and output ports are defined as waveguide ports.

III. NUMERICAL CALCULATIONS

Full wave simulation of the unit cell

We simulated the unit cell of the structure (see Fig. 1c) by means of the commercial software *CST Microwave Studio*, exciting the slot mode of the unit cell with a waveguide port. Figure S3a shows the field lines for a unit cell with periodic boundary condition in the propagation direction (*x*-direction) and electric wall boundary condition in the transverse direction (*z*-direction). This configuration produces a field distribution equivalent to that of a bi-dimensional disposition of unit cells propagating in the *x*-direction. Figure S3b shows the amplitude of the electric field versus distance along the *y*-direction. Since high order Floquet modes present shorter wavelengths (showing details in the field distribution that cannot be described by the fundamental mode), the field induced by these modes shows a higher decay ratio than the field generated by the fundamental mode. Thus, this contribution disappears rapidly from the surface of the device. This effect can be seen in lower values of distance in Fig. S3b, where the fields generated by these modes induce a deviation from the exponential decay. For distances higher than approximately 2 cm the field distribution can be perfectly described by the contribution of the fundamental Floquet mode only. Figure S3c shows the isolines of the electric field amplitude in logarithmic representation (dBV/m). These lines describe space regions where the electric field amplitude presents the same value and reveals homogeneity in field amplitude in the *z*-direction. The picture shows an equidistance of isolines in the region dominated by the fundamental mode, as is expected from the theoretical prediction of an exponential field decay.

a

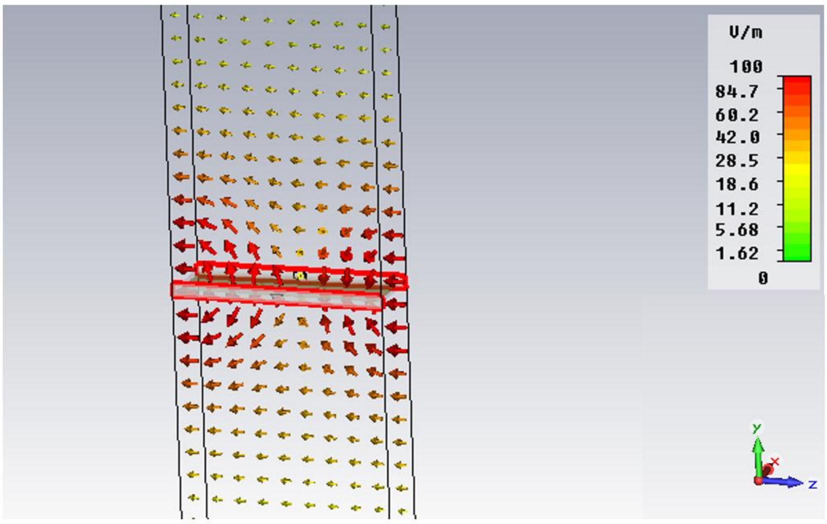

b

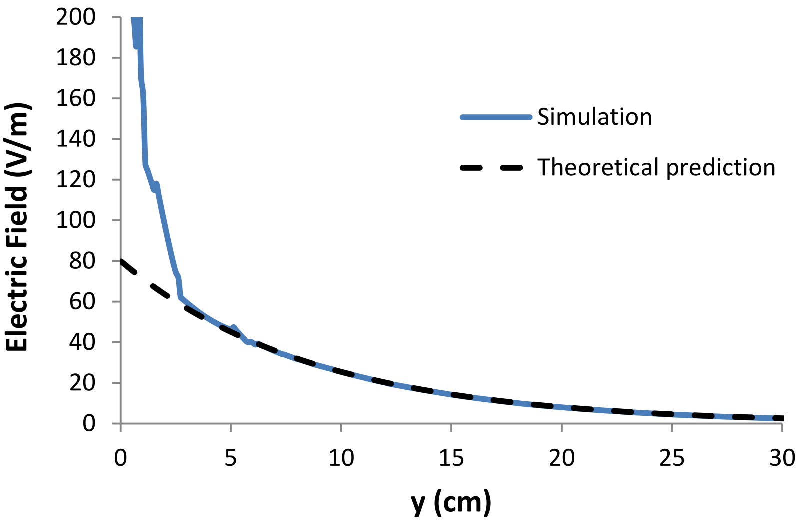

c

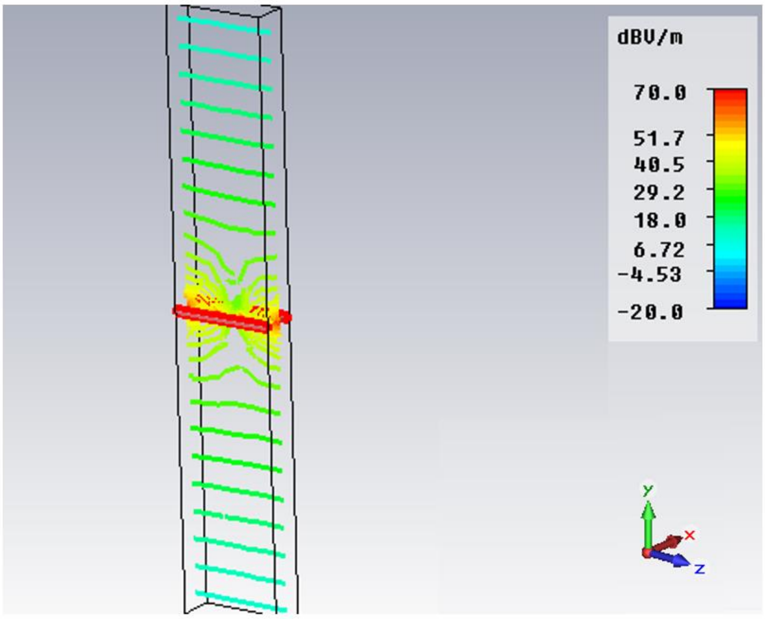

Fig. S3. Simulation of the unit cell with periodic and electric wall boundaries. (a) Electric Field pattern, (b) field amplitude with distance, and (c) isolines of the field amplitude.

Figure S4a shows the field distribution for a unit cell with periodic boundary conditions in *x*-direction and open boundaries in *z*-direction. This situation corresponds to the case of the fabricated prototype (Fig. 1) but with infinite length. Figure S4b shows the isolines of the electric field distribution in dBV/m, evidencing a reasonably flat dependence of the field intensity along the *y*-direction in the symmetry plane of the structure (*xy*-plane containing the slot of the host line, where the measurements of electric field were taken) and in the vicinity, valildating the hypothesis made in equation (S2) that assumes no *z*-variation of the fields. On the other hand, isolines reveal a cylindrical dependence of the fields in the *z*-direction within the *xz*-plane. Under this condition, the field decay fits with an evanescent wave with cylindrical symmetry, which is proportional to [15]:

$$A_i(z) \propto A_i H_1^{(1)}(j\alpha z) \tag{S6}$$

where $H_1^{(1)}(x)$ is the Hankel function of first kind, order 1 and argument x, and α is the attenuation constant of the guided mode. Figure S4c shows the field amplitude dependence on distance for both directions (y-direction is depicted in blue and z-direction is depicted in orange) and the theoretical adjustment according to equation (S5) (y-direction, depicted in dot-dashed bold line) and equation (S6) (z-direction, depicted in dashed bold line). The simulated results fit very well in both cases once the influence of higher order Floquet modes is negligible. This result ensures the confinement of the field around the structure in all directions of the space even in the case of a unidimensional configuration of unit cells.

a

b

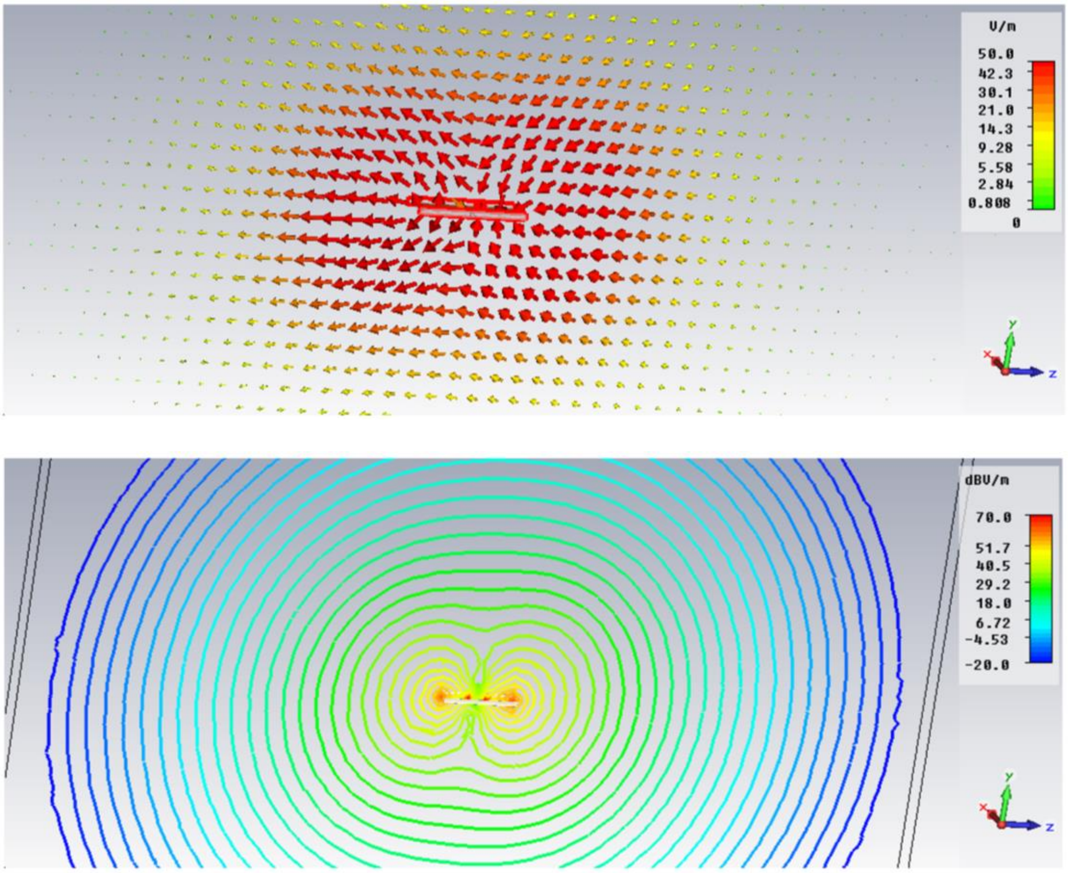

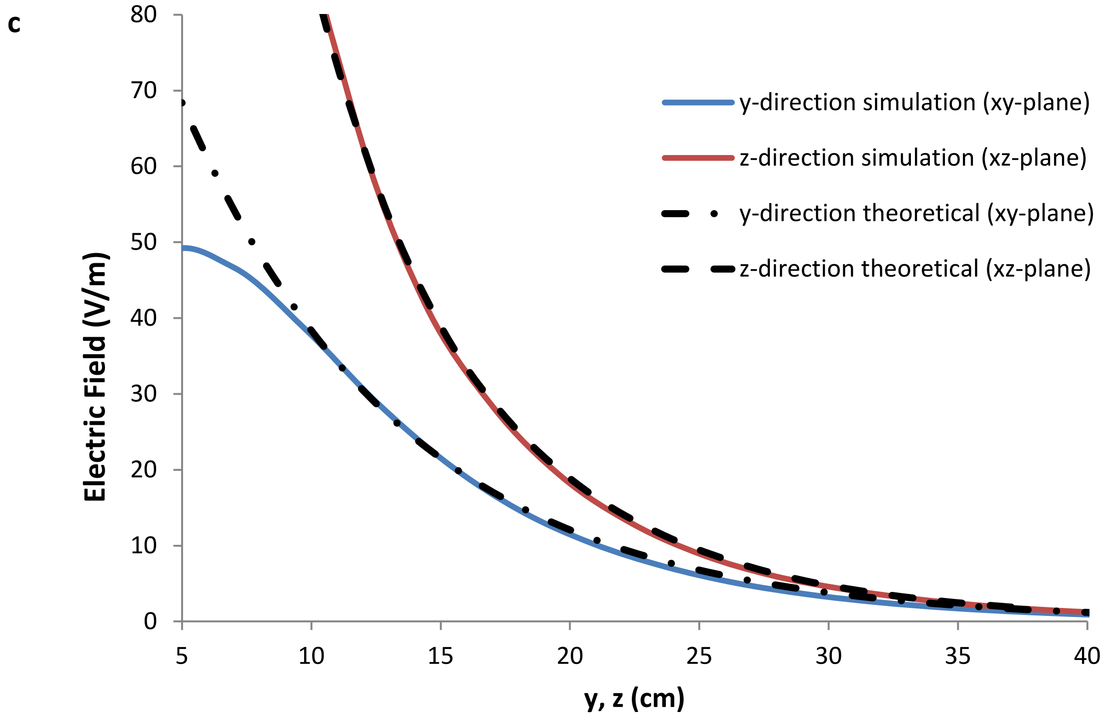

Fig. S4. Simulation of the unit cell with periodic and open boundaries. (a) Electric Field pattern, (b) isolines of the field amplitude, and (c) field amplitude with distance.

<u>Full wave simulation of the scattering parameters of the structure</u>

We simulated the S_{21} and S_{11} parameters of the structure by means of the *Keysight ADS Momentum* commercial software (see Fig. S5). A good matching to the reference impedance (50 Ω) can be observed at the operating frequency band. The simulation insertion losses are 2.7 dB at 867 MHz.

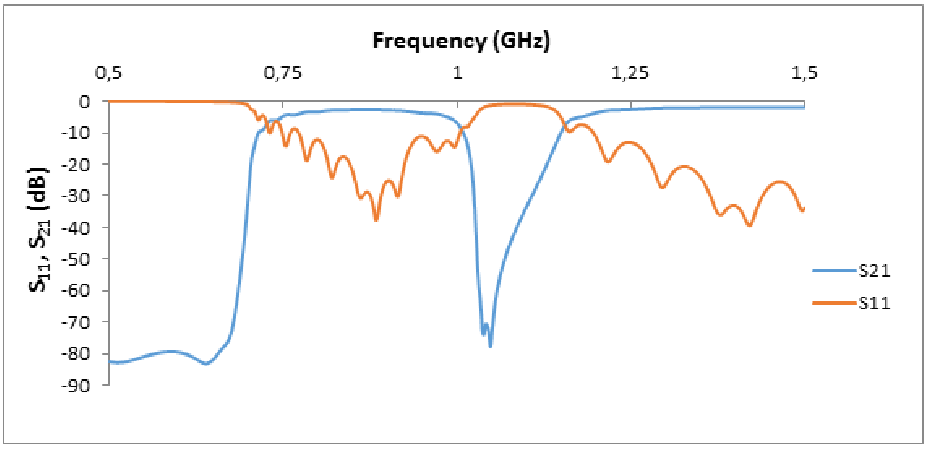

Fig. S5. Simulated scattering parameters of the device.

IV. MEASUREMENT OF THE SCATTERING PARAMETERS

Measured results are depicted in Fig. S6. Good impedance matching to the reference impedance is appreciated since the S_{11} parameter is below −20 dB at the operating frequency. The measured S_{21} parameter shows an insertion loss of 4.8 dB at 867 MHz, corresponding to an attenuation of 0.15 dB/cm. It shows good concordance with simulated results (Fig. S5) although measurements present higher insertion losses, due to the fact that simulations were performed defining the host line as a slot layer in *Momentum* (and hence considered as a lossless layer by the simulator).

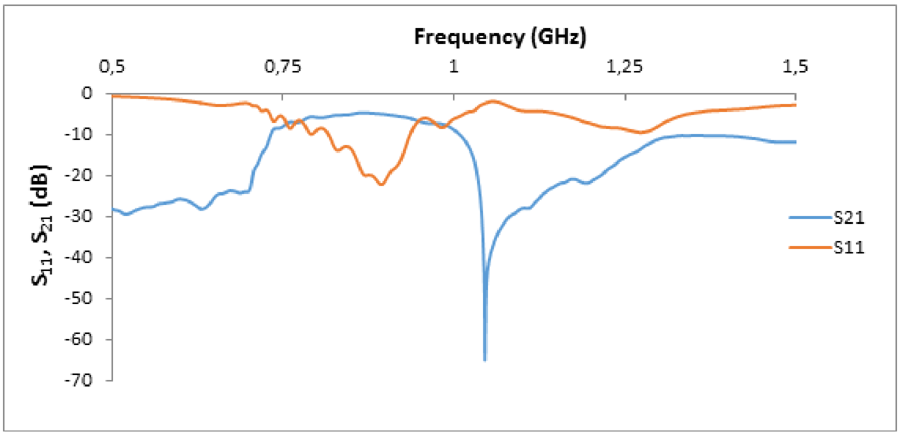

Fig. S6. Measured scattering parameters of the device.

---

1. Zucker F., J. Surface-wave antennas in *Antenna Engineering Handbook* (ed. Johnson, R.C) Ch. 12 (McGraw-Hill, NY, ed. 3, 1993).
2. Born, M. & Wolf, E. Principles of Optics (Cambridge University Press, Cambridge, ed. 7, 1999).
3. Oliner, A. Leaky-wave antennas in *Antenna Engineering Handbook* (ed. Johnson, R.C) Ch. 10 (McGraw-Hill, NY, ed. 3, 1993).
4. Jackson, D. R., Caloz , C. & Itoh, T. Leaky-wave antennas. *Proc. IEEE* **100,** 2194–2206 (2012).
5. Liu, L., Caloz, C. & Itoh, T. Dominant mode leaky-wave antenna with back fire to end fire scanning capability. *Electron. Lett.* **38,** 1414–1416 (2002).
6. Grbic, A. & Eleftheriades, G. V. Leaky CPW-based slot antenna arrays for millimeter-wave applications. *IEEE Trans. Antennas Propag.* **50,** 1494–1504 (2002).
7. Arnedo, I. *et al.* Forward and backward leaky wave radiation in split-ring resonator-based metamaterials. *IET Microw. Antenna P* **1,** 65–68 (2007).
8. Grbic, A. & Eleftheriades, G. V. Experimental verification of backward-wave radiation from negative refractive index metamaterial. *J. Appl. Phys.* **92,** 5930–5935 (2002).

9. Smith, D. R., Pendry, J. B. & Wiltshire, M. C. K. Metamaterials and negative refractive index. *Science* **305,** 788–792 (2004)
10. Smith, D. R., Padilla, W. J., Vier, D. C., Nemat-Nasser, S. C. & Schultz, S. Composite medium with simultaneously negative permeability and permittivity. *Phys. Rev. Lett.* **84,** 4184–4187 (2000).
11. Caloz, C., Chang, C.-C. & Itoh, T. Full-wave verification of the fundamental properties of left-handed materials in waveguide configurations. *J. Appl. Phys.* **90,** 5483–5486 (2001).
12. Martín, F., Falcone, F., Bonache, J., Marqués, R. & Sorolla, M. Split ring resonator based left handed coplanar waveguide. *Appl. Phys. Lett.* **83,** 4652-4654 (2003).
13. Caloz, C. & Itoh, T. *Electromagnetic Metamaterials: Transmission Line Theory and Microwave Applications* (Wiley-IEEE Press, NJ, 2005).
14. Bonache, J., Gil, I., Garcia-Garcia, J. & Martin, F. Novel microstrip bandpass filters based on complementary split rings resonators. *IEEE Trans. Microwave Theory Techn.* **54**, 265–271 (2006).
15. Cohn, S. B. *IEEE Trans. Microw. Theory Techn.* **17,** 768–778 (1969).
